# The unattainable other: a Lacanian psychoanalytic perspective on digital laborers’ traffic anxiety

**DOI:** 10.3389/fpsyg.2026.1870187

**Published:** 2026-07-29

**Authors:** Qiuyu Fan

**Affiliations:** 1 Department of Philosophy, Shanghai Normal University, Shanghai, China; 2 Institute of Marxism, Anhui Academy of Social Sciences, Hefei, China

**Keywords:** digital labor, libido, *objet petit a*, surplus jouissance, the big Other, traffic anxiety

## Abstract

“Traffic anxiety” has become a collective psychic condition among digital content producers. Drawing on Lacanian psychoanalysis as its theoretical framework, this article conceptualizes “traffic” (viewership metrics) as *objet petit a*, arguing that it is constructed under the gaze of the algorithmic “big Other” as an ever-escaping object-cause of desire. After clarifying the functional division of labor between *objet petit a* and surplus *jouissance*, it further introduces the distinction between phallic *jouissance* and the *jouissance* of the Other, providing theoretical resources for understanding different subtypes of traffic anxiety. The article then theoretically articulates five mechanisms at work in traffic anxiety: frustration anxiety arising from blocked libidinal investment, castration panic under the threat to symbolic identification, the somatization and social displacement of anxiety, repetition compulsion within a temporal structure, and the failure of defense mechanisms and deepening of self-exploitation. Rather than offering empirical validation, the study deploys a theoretical-interpretive design in which autoethnographic diaries serve as an exemplary case that renders a psychic structure legible, while findings from existing qualitative and quantitative literature are drawn upon as corroborating context. The theoretical contribution of this article lies in demonstrating that traffic anxiety reflects a deeper reorganization of the economy of desire by digital platforms—a restructuring that shapes laborers’ subjectivity at the unconscious level, extending platform exploitation beyond the appropriation of labor products to the depletion of laborers’ very libidinal production.

## 1 Introduction

In 2018, a YouTuber who had toiled for years posted a tearful farewell video with a disturbingly blunt title: “I cannot do this anymore.” She was not saying goodbye because of a lack of traffic—her channel had hundreds of thousands of subscribers—but because every refresh of the data brought immense psychological stress. Such experiences are becoming a collective symptom among digital laborers. Some scholars have already attended to this issue. However, existing research has tended to leave the laborers’ unconscious subjectivity undertheorized. Political economy critiques have made indispensable contributions by revealing the discipline and extraction of surplus value achieved by platforms through algorithmic control (Scholz, 2013; Fuchs, 2014; Wood et al., 2019). Their analytical strength, however, lies primarily in the external control dimension of the labor process, and they do not claim to account for the persistence of anxiety when economic coercion is not the immediate driver. Social-psychological approaches treat traffic anxiety as a form of social media fatigue. Bucher proposed the concept of the “algorithmic imaginary” from a critical algorithm studies perspective (Bucher, 2017). This concept takes an important step toward the unconscious dimension by showing how the unknowability of algorithms itself functions as a disciplinary force. It nevertheless remains primarily at the level of Foucauldian discipline and has not yet entered the core terrain of the economy of desire. These explanatory frameworks all presuppose a rational-choice model of the subject and struggle to handle those discourses that overflow rationality: Why does “zero likes” trigger the existential panic of “I do not exist”? Why does a creator with a million followers still feel insecure? Why is it impossible to stop refreshing data despite knowing it is agonizing?

The answers to these questions point to the laborers’ unconscious subjectivity—the subject who experiences a symbolic life and death before the platform interface and data feedback. This article introduces the theoretical tools of Lacanian psychoanalysis, especially its concepts of libidinal economy, *objet petit a*, and the big Other, in order to carry out a deep structural analysis of traffic anxiety. The core argument unfolds in three progressive propositions: (1) “traffic” is constructed as *objet petit a*—an object-cause of desire that promises complete satisfaction yet always eludes; (2) algorithms function as the embodiment of the big Other, and their data feedback constitutes a verdict on the laborers’ symbolic existential value; (3) the laborers’ anxiety is the psychic energy of libidinal investment in the big Other that has not been properly returned, which comes back upon the self in the form of symptoms and is continuously reproduced through repetition compulsion. Accordingly, this article is primarily a work of theoretical elaboration: it proposes a Lacanian translation of traffic anxiety and a five-dimensional framework for understanding its psychic operations, with autoethnographic diary entries serving as illustrative material that makes these theoretical mechanisms concretely observable. Its specific contribution lies in demonstrating that digital platforms do not merely extract labor or induce social comparison, but fundamentally reorganize the economy of desire—reshaping how subjects are libidinally invested, symbolically recognized, and psychically constituted in the digital age. The study employs a theoretical-interpretive design: autoethnographic diaries are treated as an exemplary case that makes a psychic structure observable, while quantitative and qualitative findings from the literature are integrated as corroborating material. This approach foregrounds the theoretical articulation of five psychic mechanisms of traffic anxiety, illustrated through first-person material, rather than seeking empirical representativeness. Finally, the political implications of the libidinal economy behind traffic anxiety are discussed.

## 2 Theoretical framework: traffic as *objet petit a* and the completion of the libidinal economy

### 2.1 Between Freud and Lacan: the evolution of anxiety theory

The term “libidinal economy” refers to the systemic circulation of libidinal energy—the psychic fuel of desire, investment, and satisfaction—within a given social and symbolic structure. In the context of digital platforms, it designates the regime through which laborers’ desire is mobilized, channeled, extracted, and partially returned in the form of traffic data.

To understand the psychic mechanism of traffic anxiety, it is necessary to first clarify the theoretical evolution of the concept of “anxiety” within the psychoanalytic tradition. Freud’s theory of anxiety went through two stages of development. In the early stage, Freud regarded anxiety as a direct transformation of “blocked libido”: when sexual excitation is repressed and cannot be discharged through normal channels, it seeks an outlet in the form of anxiety. Libidinal investment here refers to the psychic energy the subject directs toward an object or activity; in digital labor, it includes not only time and effort but also the narcissistic libido invested in self-presentation and the desire for recognition. When these investments cannot flow back in the form of traffic, the blocked libido is internalized as anxiety (Freud, 1895/1962; Freud, 1917/1963). In 1926, Freud proposed a second theory of anxiety: anxiety is no longer merely a metabolic waste of libido but a signal of the ego. “It would be truer to say that symptoms are created so as to avoid a *danger-situation* whose presence has been signalled by the generation of anxiety” (Freud, 1926/1959, p.129). It warns the subject of an impending danger situation, such as loss of the object, loss of the object’s love, the threat of castration, and punishment by the superego. In Lacanian terms, castration is not a physical threat but a symbolic operation: it refers to the subject’s necessary submission to the signifier, the loss of an immediate access to *jouissance* upon entering the symbolic order. Symbolic castration thus marks the subject’s constitutive lack—the price of being represented in language and recognized by the Other. Here anxiety is an anticipatory defense mechanism. In traffic anxiety, the laborer does not wait until the data are finally known to feel anxious; rather, anxiety is activated the moment the “publish” button is pressed—an early warning of “possible non-recognition.”

Lacan decisively recast this in his *Seminar X: Anxiety*. He categorically rejected the explanation that anxiety is simply a signal function, firmly anchoring the definition of anxiety to the pivotal concept of his theory of desire: *objet petit a*. The *objet petit a* is not the empirical object of desire but the object-cause that sets desire in motion; it is a remainder of the Real that can never be fully symbolized and that promises a *jouissance* which constantly eludes the subject. Lacan’s thesis is that anxiety does not arise from lack; on the contrary, it arises from the lack of lack, that is, when *objet petit a*, which is supposed to be missing, suddenly comes too close to the subject, threatening to fill the void that sustains the movement of desire. The unique feature of anxiety is that, unlike fear, it does not have a definite object; the “object” of anxiety is precisely that which cannot be objectified—the unknowable x of the Other’s desire, the suffocating approach of “*Che vuoi?*” (“What do you want from me?”) (Lacan, 2014). In Lacan’s work, the Freudian “loss of the object’s love” is re-anchored in the fundamental relationship between the subject and the Other. The panic of being abandoned by the Other is not a fear of some specific object, but a deep shudder of “no longer having a place in the Symbolic.” This panic constitutes the unconscious prototype of the “zero likes” experience in traffic anxiety: when data remain silent, the laborer encounters not merely an absence of feedback but the threat of symbolic death, of being struck from the Other’s signifying register.

Freud’s and Lacan’s models of anxiety are not mutually exclusive. In traffic anxiety, we can see their interweaving at different levels: the internalization of blocked libido explains the energy source, somatic intensity, and temporal position of anxiety; while the approach and withdrawal of *objet petit a* explains the structural logic of anxiety—why no amount of traffic can ever fill the hole, and why every refresh is both a relief and a new torment. Integrating these two anxiety models enables this article to address both the energetic and structural dimensions of traffic anxiety, avoiding the reduction of a complex psychic phenomenon to a single mechanism.

### 2.2 Surplus *Jouissance* and the capitalist discourse

To deepen the analysis of traffic anxiety, it is necessary to introduce Lacan’s discussion of *jouissance*, especially the concept of surplus *jouissance* (*plus-de-jouir*). Surplus *jouissance* is Lacan’s psychoanalytic rewriting of Marx’s concept of surplus value: just as the capitalist extracts surplus value from the labor process that exceeds the cost of labor power, the signifying system also extracts from the subject a surplus *jouissance* beyond the satisfaction of need. It is an excessive, painful-pleasurable satisfaction that the subject experiences as a loss of being. “‘What does it pay in?’ he says. ‘It pays in *jouissance*, precisely, and this has to go somewhere.’ What’s disturbing is that if one pays in *jouissance*, then one has got it, and then, once one has got it it is very urgent that one squander it. If one does not squander it, there will be all sorts of consequences” (Lacan, 2007, p. 20). The paradox of surplus *jouissance* is that it is not a pure pleasure; on the contrary, it is an excessive satisfaction mixed with suffering, in which the subject simultaneously experiences its own loss. “It begins with a tickle and ends in a blaze of petrol. That’s always what *jouissance* is” (Lacan, 2007, p. 72).

Here it is necessary to clarify the functional division of labor between *objet petit a* and surplus *jouissance*. *Objet petit a* is the object-cause of desire; it is constructed as an ever-unattainable goal, and it is precisely its perpetual absence that keeps desire flowing. Surplus *jouissance*, on the other hand, is the symptomatic precipitate mixed with pain that overflows from the subject’s own libidinal economy due to the operation of the signifying system in the process of chasing this object-cause. In the context of digital platforms, traffic, as an ideal signifier that is promised to complete the sense of existence yet never arrives, occupies the position of *objet petit a*; and the compulsive mixture of excitement and exhaustion experienced by laborers in the act of repeatedly refreshing the backend and chasing data curves, that “pleasurable pain” that one cannot stop despite knowing it is futile, is surplus *jouissance*.

Lacan further distinguished between phallic *jouissance* and the *jouissance* of the Other. Phallic *jouissance* is the *jouissance* that operates within the symbolic order, tied to signifiers and the phallic function; it can be quantified, compared, and achieved, manifesting as the pursuit of a specific data threshold and the transient satisfaction upon reaching it. The *jouissance* of the Other points to a *jouissance* beyond symbolization, outside the signifying system, a *jouissance* of the extreme often tinged with a painful bodily satisfaction (Lacan, 1998a). In the context of digital platforms, this distinction provides theoretical resources for understanding two subtypes of traffic anxiety: some laborers’ anxiety revolves mainly around an imbalance in symbolic exchange, a blockage in the symbolic economy of phallic *jouissance*. Their core anxiety is “the data is not good enough,” and their predicament can still be articulated within the symbolic order. Another group of laborers has slipped into an almost bodily “*jouissance* circuit” with the algorithm; they repeatedly go live not to pursue measurable phallic *jouissance* but to confirm, through endless scrolling and bodily exhaustion, a minimal sense of “I am still here.” This is precisely the pathological expression of the *jouissance* of the Other in the digital age.

The capitalist discourse that Lacan formulated in his later years provides a broader theoretical framework for this mechanism (Lacan, 1978, discussed in Žižek, 2009). Unlike the traditional master’s discourse, the capitalist discourse abolishes the prohibitive function of the signifier, making it seem as if the subject can directly access objects of enjoyment. But in reality, it is precisely this “abolition of prohibition” that plunges the subject into a deeper predicament: the acquisition of enjoyment is organized into an infinite cycle of consumption that is never truly satisfied. Žižek articulates the fundamental logic of the capitalist discourse as the “superego imperative to enjoy”—“The superego imperative to enjoy thus functions as the reversal of Kant’s *‘Du kannst, denn du sollst!*’ (You can, because you must!); it relies on a ‘You must, because you can!’” (Žižek, 2009, p. 58). That is exactly why you can never feel you have enjoyed enough. Digital platforms, with their promises that “you can have a million followers” and “you can become famous overnight,” annihilate the traditional symbolic order’s restrictions on the acquisition of signifiers, throwing everyone into the arena of competing for traffic. Platforms lock the laborers’ libido permanently onto the track of continuous optimization and relentless investment, while the traffic return from each “optimization” is calibrated precisely to an amount sufficient to keep desire running but never enough to allow it a moment of peace. It disciplines not through prohibition, but by throwing the subject into a field of choice that appears free but is in fact pre-structured, thereby extracting its libidinal energy.

### 2.3 The big other, the symbolic, and the algorithmic verdict

In Lacanian theory, the Other (*Autre*) is not a specific person or institution, but the locus of the symbolic order, the signifying system constituted by all signifiers that provides coordinates and recognition for the subject (Lacan, 2006). The subject must seek recognition in the field of the Other—individuals’ sense of existence, value, and identity are all constructed and maintained in relation to the Other. The most fundamental characteristic of the Other is its unknowability: the subject can never know with certainty what the object of the Other’s desire is, and can only engage in endless conjecture based on the signifying feedback the Other gives (Lacan, 1977). In the field of digital labor, the algorithm occupies the structural position of the Other. The algorithm maintains the ledger of all signifiers—data—and passes judgment on each subject. It decides which content “deserves” to be seen through the distribution of traffic pools, determines the laborers’ visibility fate through the raising and lowering of recommendation weights, and keeps laborers in a perpetual state of “divining the will of the sovereign” through obscure rule changes. Admittedly, the algorithm is code written by engineers and has a material basis. But on this side of the interface, for the laborer facing the screen, the algorithm’s operation exhibits all the fundamental characteristics of the Other: it is silent, opaque, omniscient, and capricious. Just as the question of the Other’s desire is “*Che vuoi?*”—so too do the laborers ask: What does the algorithm want? Unlike human authorities, the algorithmic Other does not even have a readable “facial expression”—it only outputs numbers, and the meaning of these numbers must always be guessed by the laborers themselves.

The algorithmic verdict carries such immense psychic power because it is experienced as a distribution of symbolic existence. In the platform economy, traffic is not merely economic remuneration; it is first and foremost a signifier that marks the subject’s existential weight in the Other’s ledger. “100 k reads” means not only a share of advertising revenue, but more fundamentally “you are important, you are recognized, you exist in the public eye.” Conversely, zero likes or being shadowbanned is experienced at the unconscious level as a symbolic death—the real trauma signified by “nobody sees what I post” is not a loss of economic benefit, but the panic of “I no longer occupy a visible position in the world.” In other words, what laborers desire is not traffic per se, but the sense of existence that traffic, as a signifier, guarantees for them in the field of the Other. The quantifiability of this existence is the core feature of the crisis of subjectivity in the digital age: existence becomes not a philosophical question, but a data question.

### 2.4 Analytical framework of this article

Based on the theoretical construction above, this article proposes a multidimensional analytical framework to interpret the psychic operations of traffic anxiety. The framework consists of the following five interrelated analytical dimensions:

#### 2.4.1 Libidinal investment and its blockage

Drawing on Freud’s first theory of anxiety, this dimension examines how laborers invest libidinal energy in content production and how, when the return of traffic is insufficient, the blocked libido is internalized, forming frustration-type anxiety.

#### 2.4.2 Threat to symbolic identification and castration panic

Drawing on Lacan’s theory of anxiety and the concept of the big Other, this dimension examines how negative fluctuations in traffic data are experienced as symbolic castration of the subject—the revocation of their signifying credentials—triggering a panic that touches the foundations of existence.

#### 2.4.3 Somatization and social displacement of anxiety

This dimension traces how anxiety overflows from the purely psychic level, transferring into the body (insomnia, psychosomatic symptoms) and social relations (conflict, alienation), revealing the invisible bodily costs of the libidinal economy.

#### 2.4.4 Temporality and repetition compulsion

The Freudian concept of the death drive—the compulsion to repeat beyond the pleasure principle, a tendency toward a return to an inorganic state that manifests psychically as repetitive, self-destructive patterns—is central to this dimension. It reveals why laborers are locked in a temporal prison where “the future is always elsewhere,” and the drive economics behind the compulsive behavior of repeatedly refreshing data.

#### 2.4.5 Defense mechanisms and self-exploitation

In psychoanalytic theory, defense mechanisms are unconscious strategies deployed by the ego to manage anxiety by keeping threatening impulses, affects, or ideas out of conscious awareness. They operate not by resolving the underlying conflict but by displacing, denying, or transforming it—often at the cost of a deeper entrenchment of the very structure they seek to manage. Employing Lacan’s concepts of surplus *jouissance* and the capitalist discourse, this dimension examines how laborers’ various defensive strategies against anxiety—from compulsive checking to cynicism—paradoxically deepen self-exploitation and trap them in the cycle of desire.

These five dimensions are interdependent and form a dynamic continuum: the first two explain the sources of anxiety from the perspectives of energy economics and symbolic identification respectively; the third and fourth reveal the diffusion paths and temporal structure of anxiety; the fifth dimension demonstrates the (ineffective) coping strategies of laborers and their deeper consequences.

This five-dimensional framework constitutes the article’s primary theoretical contribution: while each dimension draws on established psychoanalytic concepts, their integration into a unified account of digital traffic anxiety—and the articulation of their interrelations as a dynamic continuum—is proposed here for the first time.

## 3 Research design: a theoretical-interpretive approach with autoethnographic illustration

### 3.1 Epistemological positioning and overall design

This study does not seek statistical representativeness or empirical hypothesis testing. Its aim is to make a psychic structure—the libidinal economy underlying digital labor—visible through theoretically guided interpretation. The epistemological standard proper to psychoanalytic inquiry is interpretive coherence and clinical plausibility, not falsifiability in the positivist sense. In this tradition, a single case can function as an exemplary prism through which a structure becomes legible, analogous to the psychoanalytic clinical vignette. The study accordingly adopts a theoretical-interpretive design: autoethnographic diaries provide privileged first-person material that renders subjective dynamics observable, while published qualitative and quantitative findings are integrated as corroborating context rather than as objects of a systematic empirical test.

### 3.2 Use of existing literature

The literature cited in Section 4 serves two distinct roles. Quantitative studies (e.g., Meier and Schäfer, 2018; Burnell et al., 2019; Feltman and Szymanski, 2018) are drawn upon to establish patterns of association—between likes and self-worth, passive use and depressive symptoms, self-objectification and relational conflict—that the psychoanalytic framework reinterprets at the level of unconscious structure. Qualitative studies (e.g., Lou and Zhou, 2024; Duffy et al., 2021; Cotter, 2019) are treated as providing first-person narratives and thematic findings that are reread through a Lacanian lens. At no point are effect sizes, mediation paths, or survey variables subjected to a symptomatic reading in the Althusser-Macherey sense; such reading is reserved for the narrative and autoethnographic material where gaps, contradictions, and silences can be discerned. The literature was identified through a targeted search of major databases (e.g., Web of Science, Scopus, PsycINFO) from 2015 to 2025, using keywords related to social media, digital labor, anxiety, and well-being, and selected for their relevance to the psychic dimensions theorized in Section 2.

### 3.3 Autoethnographic diary

The researcher, who has been part-time content creating on Bilibili and rednote since 2024, has long experienced traffic anxiety from a first-person position. From September to December 2025, bodily sensations, emotional fluctuations, and behavioral impulses after each content publication were recorded in the form of field diaries, accumulating 57 diary entries. The diary adopted a dual-layer structure of “immediate recording + re-evaluation after a 24-h interval”: original feelings and bodily reactions were recorded immediately after publishing, and self-analysis was supplemented 24 h later through rational reflection. This autoethnographic material does not serve as evidence of prevalence; it operates as an exemplary case that makes a psychic structure concretely observable. Throughout the article, autoethnographic accounts are marked as “Researcher’s Diary” with the entry number.

While the dual-layer structure was designed to create distance between raw experience and theoretical interpretation, the researcher’s Lacanian commitments inevitably inform both layers; the diary is offered not as a theoretically neutral record, but as material that is already shaped by—and in turn illuminates—the framework it serves.

All diary entries document the first author’s personal experiences as a content creator; no third-party data or identifiable information is included. As the researcher is also the sole participant, formal institutional ethics review was not required; nevertheless, the dual-layer recording structure was designed to respect the temporal boundary between lived experience and analytical reflection.

### 3.4 Platform Interface walkthrough

From September to December 2025, the researcher conducted structured walkthroughs of the data dashboards in Douyin’s Creator Center and rednote’s Creator Center (Light et al., 2018). These platforms were selected because they collectively cover short-video, image-text, and mid-length video formats, capturing the dominant content ecologies of the Chinese digital labor market. The analysis focused on how the interfaces generate a sense of urgency and desire through color, numerical presentation, fluctuation curves, and push notifications, as well as how animation design induces compulsive checking behavior. These observations inform the interpretation of how the algorithmic Other materially inscribes itself on the laborer’s experience but are not analyzed as independent data.

### 3.5 Integration and analytical procedure

The core of the analysis in Section 4 involves a hermeneutic movement between theoretical concepts and autoethnographic narrative. Diary entries were openly coded for recurring themes (e.g., “judgment,” “cannot stop,” “subconscious checking”). Coding was performed manually through repeated reading of the diary entries, with emergent themes cross-checked against the five-dimensional framework articulated in Section 2.4. The resulting themes were then interpreted through that framework. The goal is not to prove that the five mechanisms exist in a population, but to demonstrate their internal coherence and their capacity to illuminate subjective experiences that remain opaque under other explanatory frameworks.

## 4 Illustrative analysis: a symptomatic reading of the five psychic mechanisms

This section deploys the five theoretical dimensions to interpret the subjective dynamics of traffic anxiety, drawing primarily on the autoethnographic diary and secondarily on published narratives and quantitative findings as corroborating context. The analysis is illustrative rather than empirically validating; it aims to show how a Lacanian libidinal economy makes sense of experiences that exceed rational-choice or political-economy accounts. The five dimensions are presented as an articulated continuum, with each shading into the next.

### 4.1 Libidinal investment and its blockage: from like thresholds to anxious conversion

An experiment by Meier and Schäfer (2018) revealed that Instagram users instantaneously translate the number of likes into a frame of reference for self-worth—low likes triggered a significant experience of self-depreciation. While this quantitative finding establishes an association, the psychoanalytic framework reinterprets it as evidence of a libidinal exchange: in content production, laborers invest the courage of self-exposure, time spent on aesthetic refinement, and desire for recognition—elements of libidinal energy that cannot be directly quantified. These need to be symbolically confirmed upon return through the number of likes. When the threshold is not met, the blocked libido finds no external channel for discharge and implodes into somatic symptoms.

During the backend walkthrough, the researcher noted that Douyin’s Creator Center often uses a bright orange color to prominently display comparison metrics between a creator’s content and that of their peers, for instance, showing whether a post’s performance is below the average for similar content. After one post, the researcher’s diary recorded:

“15:32, published. 16:00, 87 views, 5 likes. I stared at that grey bar—it told me ‘views lower than 80% of similar posts’. My chest began to tighten. I know this number has no actual consequence, but the ‘80%’ felt like a judgment. What it said was—your position is in the bottom tier. Your existence is in the bottom tier.” (Diary Entry #3)

The interface here performs multiple functions: transforming “insufficiency” from a subjective feeling into an objective ranking, creating urgency through color coding, and anchoring the gaze of the big Other in every refresh through real-time data updates. Particularly noteworthy is the metaphor of “judgment”—it is not a random rhetorical flourish, but a precise unconscious designation of the algorithmic Other’s verdict function.

Research by Abidin (2016) on influencers in Singapore reported that they must continuously engage in “careful self-presentation,” opening themselves up to non-stop quantitative metrics. The researcher’s diary also confirmed this ceaselessness and the deep sacrifice implicit in it—entry #34 records the compulsive sense of “cannot stop”:

“Not feeling well today, wanted to take a day off from posting. But at 4 p.m. I still opened the editing software. It wasn’t that someone forced me; it was that thought—‘miss one day, and the algorithm will forget you’—stuck in my head. After posting, I kept brushing the data, same as always, neither high nor low. It seems the only way to lessen the anxiety is exactly what creates the anxiety in the first place.” (Diary Entry #34)

The last sentence pinpoints an ontological reversal: the only way to diminish anxiety is precisely to generate it. Using labor itself to alleviate the anxiety about “not laboring”—this paradoxical structure indicates that compulsive content production is not a positive pursuit of desire, but a negative defense against the fundamental fear of “absence equals disappearance.”

### 4.2 The threat to symbolic identification: traffic failure as a castrating experience

If blocked libido triggers anxiety that is reversible, then the sense of self-disintegration triggered by a continuous slump in traffic data is even more lethal—it touches the very foundation of the subject’s occupation of a signifying position in the Symbolic.

In a study on female social media users’ envy, Chae (2018) observed an association whereby when exposed to the “idealized” content of influencers, the perceived self-other discrepancy significantly positively predicted envy and negative self-evaluation. Interpreted psychoanalytically, this negative self-evaluation not only targets specific content performance but also permeates the overall self-concept—when “my content is not good enough” transforms into “I am not good enough,” the subject crosses the critical threshold from external attribution to collapse of self-identity. This marks the moment when castration anxiety shifts from “fear of symbolic deprivation” to “negation of the essence of the self,” a shift that Freud (1917/1963) theorized under the concept of melancholia, where the shadow of the lost object falls upon the ego.

Burnell et al. (2019), in a study on adolescents’ passive social media use, provided findings that can be read as a more detailed empirical depiction of this self-identity collapse. The study revealed that passive use indirectly predicted depressive symptoms and a decline in overall self-worth, fully mediated by social comparison and fear of missing out. The researchers particularly pointed out that the negative self-evaluation under the “silent consumption” model was not a direct reaction to external feedback, but “spontaneous melancholy” produced by users after repeatedly measuring the self-other gap internally. From a psychoanalytic perspective, this spontaneous melancholy is precisely the experiential portrayal of the melancholic position in the context of social media: unlike mourning—where, despite the pain, the subject can still separate the lost object from the self—the subject in melancholy turns the anger against the lost object entirely upon the self (Freud, 1917/1963).

The researcher’s diary entry #21 illustrates a complete descent from external attribution to the melancholic position after a data slump:

“The video I posted last night still has not broken a thousand views. I went to look at another creator in the same niche—her video broke ten thousand in just two hours. My first reaction wasn’t ‘her content is better,’ but ‘I’m not worthy of doing this.’ Then I started recalling all the videos with poor data, listing them one by one, as if compiling a list of evidence against myself. By the time I realized what I was doing, it was already two in the morning.” (Diary Entry #21)

The metaphor of a “list of evidence” is a precise symptomatological marker of the melancholic state—it indicates that the subject has already interpreted the traffic data as courtroom evidence of their own “unworthiness to exist,” and has thoroughly internalized the Other’s verdict as a self-conviction. From a Lacanian perspective, this diary entry documents the experience of “shadowbanning”: in the Symbolic, being shadowbanned is equivalent to having one’s name crossed out of the signifying register, and the subject’s reaction is not anger, but self-incrimination—precisely the typical symptom of melancholy. The platform’s practices of “shadowbanning” and “de-platforming” re-enact, at the operational level, an original symbolic castration: you are deletable; the big Other does not need you.

### 4.3 Somatization and social displacement of anxiety: from digital Interface to body and relationships

When this threat of symbolic annihilation cannot be metabolized at the level of signification, it presses beyond the symbolic register into the body and into the laborer’s closest relationships—the terrain to which the analysis now turns.

Psychic anxiety never exists in a purely abstract form—it seeks pathways into the body and material relationships. Lou and Zhou’s (2024) in-depth interview study with social media influencers revealed that the relentless pace of content production has exacted a significant physical toll on creators—interviewees generally reported sleep disturbances, anxiety attacks, and somatic symptoms, with their physical and mental health systematically impaired by industry pressures. Deuze (2025), based on surveys and interviews across multiple media industries, further unveiled the structural paradox of this predicament: in media creative work, it is precisely the creation itself, which makes workers feel “love” and “excitement,” that becomes the root cause of their physical and mental depletion. This paradox—where the source of passion is also the source of exhaustion—echoes the closed loop in diary entry #34: “the only way to lessen the anxiety is exactly what creates the anxiety in the first place.” Creators are swept into an endless cycle, forced to oscillate repeatedly between continuous creation and physical-mental exhaustion, with the unpredictability of algorithms—through traffic fluctuations and changes in recommendation weights—constituting the core driving force that produces this structural uncertainty.

During the platform walkthrough, the researcher observed that the pull-down refresh design of the data button is accompanied by haptic feedback, and the red notification dot at the top of the page is widely referred to by users as a “desire trigger.” Diary entry #8 records the direct penetration of this interface design into bodily rhythms:

“I know perfectly well that turning off notifications can reduce anxiety, but I just cannot do it. Because I’m afraid—afraid of missing that ‘99+’ moment. That moment feels too good, but right after, I fall into an even deeper emptiness. It’s like a physiological addiction. It’s not that the mind wants to refresh; it’s that my finger finds its way to that red dot on its own. By the time I realize it, I’m already refreshing again.” (Diary Entry #8)

“My finger finds its way to that red dot on its own”—this expression describes with extreme precision the characteristic of the drive bypassing the conscious ego and operating directly at the bodily level. Lacan, when discussing anxiety, once said that anxiety is the moment when one “cannot see oneself in the mirror.” Diary entry #8 records a similar bodily enactment: the autonomous action of the finger signifies a rupture between the conscious I and the bodily I.

Simultaneously, blocked libido is also displaced into social relations. Feltman and Szymanski (2018) found that the process of self-objectification not only affects internal self-cognition but also significantly alters patterns of interpersonal interaction—an excessive sensitivity to social feedback makes users more prone to alienation in interactions with intimate partners. Interpreted from the perspective of libidinal economy: the libidinal energy that should have been recovered through traffic feedback is, in the absence of feedback, “short-circuited” into the closest relationships, causing irreversible losses, and the only remaining object of compensation after the loss is still that silent data screen—forming a closed, deadly loop: data does not respond → emotions are displaced into intimate relationships → relationship conflict → a lonelier return to the data screen.

### 4.4 Temporality and repetition compulsion: locked into the “eternal present-future”

The somatization and social displacement traced above unfold not in open-ended time, but within a temporal rhythm dictated by the platform—a rhythm that locks the laborer into what can be called an eternal present-future. This temporal structure constitutes the fourth dimension of traffic anxiety. The laborers’ compulsive attention to data exhibits a strict temporal rhythm: before posting, the moment of posting, the first few hours after posting, 24 h, 48 h… These nodes are not naturally formed but are framed by the temporal windows of the platform’s algorithmic recommendation mechanism. More importantly, all the laborers’ imaginings of the future condense upon the “next post”—if this one does not work, there’s always the next one. The data of this round does not end the waiting, but merely inaugurates a new cycle of investment-waiting-anxiety.

Duffy et al.’s (2021) “nested precarities” framework points out that creators need to maintain a constant frequency of content output across multiple platforms to please the algorithms, and any interruption could lead to a cliff-like drop in visibility. The laborers’ temporal experience is thus thoroughly reshaped: it is no longer “I am using time to produce content,” but “time is being colonized by the rhythm of the algorithm.”

The researcher’s diary entry #12 illustrates the real-time operation of repetition compulsion:

“In the first hour after posting, I check every five minutes. If the data curve is not steep, I start to get anxious. I know it’s more rational to wait 24 h, but I just cannot wait. Every refresh after those five minutes is a fantasy of ‘maybe this time it will be different,’ and every letdown is a micro-trauma. But I do not stop—because what if the one time I stop is exactly the time ‘this time it’s different’? This thought always wins.” (Diary Entry #12)

“This thought always wins”—its structure lies in the fact that the refreshing behavior is not because the laborer believes “next time will be different,” but because the logic of “what if” is statistically unfalsifiable. No matter how many consecutive failures, the next time could possibly be the first success. This unfalsifiability grants the thought an eternal victory—it requires no factual support, only a logical gap of possibility. This is the Freudian repetition compulsion (Freud, 1920/1955) in its digital-age symptomatic form: it is not a simple repetition of the same painful experience, but the repeated manufacturing, in every refresh, of a miniature fantasy that “it could be different,” using each “maybe this time it’s different” to confirm the necessity of the next refresh, and each letdown becomes the driving force for the next refresh. Ultimately, the laborer is locked into a temporal rhythm dictated by the algorithmic big Other, making endless investments toward a “point of satisfaction” that never arrives, consuming their fundamental life energy in repetition compulsion.

### 4.5 Defense and self-exploitation: from compulsive checking to the cage of cynicism

It is against this temporal lock-in—the endless cycle of investment, waiting, and micro-trauma—that laborers develop the defensive strategies examined below. Faced with persistent structural anxiety, digital laborers are not purely passive victims. They develop a series of defensive strategies—attempts at the level of consciousness to manage anxiety and protect the remnants of their narcissism. However, psychoanalysis repeatedly tells us that defense mechanisms often replicate, in more covert forms, that which they attempt to defend against.

Feltman and Szymanski (2018) found that Instagram users tend to adopt proactive content adjustment strategies when facing data pressure—changing posting habits, optimizing content presentation, and imitating the styles of high-like accounts. These strategies appear more “rational” in the short term, but their essence is to make users invest more deeply in the logic of self-optimization and aesthetic labor, leading in the long run to a higher level of self-objectification and emotional disturbance. This finding illustrates the paradox that the most common “solutions” are precisely the most effective ways for the symptom to sustain itself.

Examining these strategies from a psychoanalytic perspective, one can identify several classic defense mechanisms. Denial is the most common and also the easiest defense for the self to see through. The researcher’s diary entry #15 claimed “I really do not care about the data anymore,” but twenty days later, entry #35 recorded: “Woke up at two in the morning, subconsciously reached for my phone to open the backend. Views had gone up by 30, and I breathed a sigh of relief. Then I realized what I was doing—I was sighing in relief for a number I claimed I did not care about.” This is the classic operation of denial: anxiety is pushed out of consciousness but continues to accumulate in the unconscious—at two in the morning, when conscious vigilance drops to its lowest, the repressed desire breaks through in the bodily action of “subconsciously reaching for the phone.”

Reversal attributes failure to “the algorithm does not know a good thing,” providing temporary protection to narcissism, but each press of the publish button is an unconscious reaffirmation of the algorithm’s authority—one is still begging it to judge one’s value again.

Sublimation manifests as the claim that “I create for art,” but diary entry #49 exposes the self-deception within: “I told myself this video was for self-expression. But when I was choosing the title, I still gave up the more literary one and chose ‘5 Shocking Truths’—because its click-through rate is higher. I was making a decision for traffic, and then comforting myself with ‘art needs to be seen.’”

The most structurally significant defense is identification with the aggressor, a concept originating in Anna Freud’s work on the ego’s defensive operations. Cotter (2019), in a study on Instagram influencers, revealed that creators generally view understanding and adapting to the algorithm as a professional skill—they invest significant time studying the algorithm’s operating patterns and regard “playing the algorithm” as a core marker of professional competence. This behavior of actively internalizing the algorithm’s rules as the logic of one’s own desire is the classic operation of identification with the aggressor: the subject no longer asks “what gives the algorithm the right to decide my visibility,” but shifts their attention entirely to “how can I better please the algorithm.”

It may be objected that treating sustained attention, professed indifference, sublimation, and identification with the aggressor as variations on the same underlying structure renders the framework unfalsifiable—every observable behavior seems to confirm the theory. This objection, however, mistakes the level at which the psychoanalytic claim operates. The claim is not that all defensive behaviors are empirically equivalent, but that they share a structural function: they manage anxiety at the level of conscious strategy while leaving the coordinates of desire—the fundamental fantasy that “traffic will satisfy me”—intact. Differentiating among these defenses matters precisely because it reveals the multiple pathways through which the same libidinal economy reproduces itself. The standard for evaluating this reading is not whether a given behavior could hypothetically be read otherwise, but whether the reading illuminates subjective experiences—the two a.m. phone check, the self-deception of the clickbait title—that remain opaque under rational-choice or political-economy frameworks.

The shared tragedy of all these defenses is that they operate at the level of desire—adjusting goals, modifying objects, whitewashing motives—without ever touching the very structure of desire. The true meaning of Lacan (1998b) “do not give way on your desire” is this: faced with the fundamental fantasy that organizes your desire, do not evade recognizing its emptiness. The more digital laborers strive to optimize strategies, adjust mindsets, and convince themselves that they “do not care,” the more actively they submit to the reign of that fundamental fantasy—“if only I reach X, traffic will satisfy me.”

## 5 Discussion: the political implications of the libidinal economy

### 5.1 From surplus value to surplus *Jouissance*: the unconscious dimension of capitalist exploitation

The analysis in this article points toward a critical dimension deeper than traditional political economy: the exploitation of laborers by digital platforms has extended into the unconscious realm, and what is being extracted is not only labor power but the very production of libidinal energy.

Marx revealed how capital appropriates surplus value by extending labor time and intensifying work. In the field of digital content production, this mechanism undergoes a key deformation: laborers are required to contribute not only time and skill, but also desire, narcissism, emotional sincerity, and existential longing. Every moment a publish button is pressed on a platform is an act of libidinal investment. The platform, as the big Other, sustains the continuity of the libidinal flow through meticulous regulation of algorithmic returns—sometimes generous, sometimes miserly, sometimes entirely silent. The libidinal energy invested by laborers is partially returned in the form of traffic data, with the amount of return precisely calibrated at a level that “just maintains desire, but never enough to fill the lack.” The surplus between return and insufficiency—surplus *jouissance*—is appropriated by the platform capital without compensation, transformed into increased user time, additional ad displays, and rising stock prices. This is not a rhetorical adaptation of Marx, but a structural interpretation of the extraction logic of contemporary digital capitalism.

The capitalist discourse directly sutures the subject’s desire to the objects of enjoyment offered by the market, abolishing the dimension of prohibition in the traditional symbolic order—the subject is no longer “forbidden” to enjoy, but is “commanded” to enjoy. Digital platforms, through traffic incentives, organize the laborers’ libido into a sustainable “desire supply system.” Duffy et al. (2021) “nested precarities” also empirically corroborate this judgment: the emotional exhaustion of creators is not accidental, but an inevitable outcome of the platform’s continuous activation of the libidinal cycle and the overdraft of laborers’ physical and mental capacities.

### 5.2 The loss of negativity and separation as a way out

In The Burnout Society, Han (2015) diagnoses the achievement society’s mobilization mechanism of “You can,” whereby the “violence of positivity” plunges the subject into deeper burnout. However, his “achievement subject” presupposes a rationally active chooser, which cannot explain why laborers still cannot break free after seeing through the mechanism of self-exploitation. Freud’s late clinical concept of “refusal” (*Verweigerung*) describes the subject’s negative posture in the face of analytical demands—I know you want my investment, but I say “No” to you. Yet, in the platform context, this posture of refusal is extremely fragile. The illustrative analysis of this article has amply demonstrated that attempts to “interrupt” the cycle by deleting data tracking apps inevitably lead back to the cycle of desire.

The concept of separation (*séparation*) proposed by Lacan in *Seminar XI*—an act by which the subject extracts themselves from the labyrinth of the Other’s desire—offers a more theoretically grounded path for thinking about a way out, because separation targets not a behavioral-level “stopping of investment,” but a structural displacement at the level of the coordinates of desire (Lacan, 1998b; Žižek, 1989). Separation is not denying desire, nor is it ceasing investment; it is recognizing that the big Other is also lacking, and that traffic data cannot truly answer the question “are you worthy of existence?” The realization of separation does not lie in successfully “quitting the internet,” but in an irreversible shift in the subject’s coordinates of desire—traffic data may still provoke anxiety, but it can no longer define the fundamental question of “whether I exist.”

The researcher’s diary entry #55 captures a moment that gestures toward such a shift:

“One day I posted something and deliberately did not open the backend for a whole day. When I opened it in the evening, the data was very mediocre. But I found out—I was still alive, not dead. That ‘not dead’ feeling was very strange, as if I had originally thought I would die, but did not.” (Diary Entry #55)

This moment does not represent a success story of “overcoming anxiety”; rather, it illustrates a possible subjective position: after a shift in the coordinates of desire, anxiety does not disappear, but it no longer leads to the panic of “I am annihilated.” It marks a way for laborers to coexist with a desire that is never fully satisfied, without being disciplined into the extraction of surplus *jouissance*. Of course, the possibility of such a shift is highly constrained. In an economic structure that rapidly recuperates all negativity as content potential—where even “I quit the internet” becomes a viral topic—sustaining separation requires enduring the silent punishment of the Symbolic. Perhaps this is what Lacan meant by “do not give way on your desire”: not never giving up the chase, but never giving up the questioning. Is your desire truly chasing what you yearn for, or are you offering up all your libido to a fantasy that can never be redeemed?

### 5.3 Limitations

Several limitations should be acknowledged. First, the autoethnographic material derives from a single researcher operating within a specific cultural and platform context (Chinese platforms Bilibili, rednote, and Douyin), and the psychic dynamics illustrated may not transfer directly to other platform ecologies or cultural settings. Second, the Lacanian framework employed here represents one among several possible psychoanalytic approaches, and other traditions (e.g., object relations, Jungian) might foreground different dimensions of the experience. Third, the illustrative design does not permit causal inferences or prevalence estimates; its purpose is to render a psychic structure visible rather than to test hypotheses. Fourth, although the diary’s dual-layer structure (immediate recording and 24-h re-evaluation) was intended to mitigate retrospective rationalization, autoethnography remains an interpretive practice, and the researcher’s theoretical commitments inevitably shape the narrative. Finally, the qualitative and quantitative literature drawn upon as corroborating context was identified through a targeted rather than a systematic review, and relevant studies may have been omitted. These limitations do not undermine the theoretical contribution, which lies in conceptual translation rather than empirical generalization, but they delineate the boundaries within which the analysis should be read. The theoretical articulations developed here are situated within a specific platform and cultural context; their applicability beyond that context remains a question for further comparative inquiry.

## 6 Conclusion

Taking the Lacanian libidinal economy as its core framework and adopting a theoretical-interpretive design with autoethnographic illustration, this article has articulated and illustrated the psychic operation mechanisms of digital laborers’ traffic anxiety. At the theoretical level, it has conceptualized “traffic” as *objet petit a*, conceptualized the algorithm as the big Other, clarified the functional division of labor between *objet petit a* and surplus *jouissance*, and introduced the distinction between phallic *jouissance* and the *jouissance* of the Other. Taken together, these conceptual moves demonstrate how digital platforms reorganize the economy of desire and the constitution of subjectivity at the unconscious level—a contribution that extends beyond existing accounts of social comparison or capitalist exploitation. This theoretical translation allows traffic anxiety to be elevated into a psychoanalytic symptom of the crisis of subjectivity under digital capitalism, proposing that platform exploitation has penetrated the unconscious dimension—not only appropriating the products of labor and labor time, but also depleting the very production of laborers’ libidinal energy. Through the illustrative reading of autoethnographic material and the integration of existing literature, the article has exhibited the five psychic dimensions of traffic anxiety as an articulated continuum: frustration-type anxiety, castration panic, somatization, repetition compulsion, and the predicament of defense mechanisms.

The practical implications of this article do not lie in proposing specific “solutions.” If the root of traffic anxiety lies in the desire economy constructed by the platforms themselves, then an effective response must point toward the structural transformation of this economy: redesigning feedback mechanisms to weaken visible data comparisons, granting laborers greater control over their labor data, establishing transparency review systems for algorithmic recommendation mechanisms, and, on a fundamental level, reflecting on the civilizational logic of equating human existential value with digital signifiers.

Finally, let us return to the tearfully departing YouTuber at the beginning of this article. What did her tears contain? Not weakness, nor a lack of stress tolerance, but the fact that her body, at a certain limit moment, spoke the truth that her unconscious had long known but her consciousness refused to acknowledge: the confirmation of “you are worthy of existence,” perpetually deferred by the algorithmic big Other, will never arrive once and for all in the form of traffic data. Her tears are the only honest response to this structural deception.

## Data Availability

The original contributions presented in the study are included in the article/supplementary material, further inquiries can be directed to the corresponding author/s.
